# The Effect of Statins in Cancer Risk Reduction in Patients on Dialysis: A Population-Based Case-Control Study

**DOI:** 10.3390/jcm10235602

**Published:** 2021-11-28

**Authors:** Po-Huang Chen, Hong-Jie Jhou, Chi-Hsiang Chung, Cho-Hao Lee, Yi-Ying Wu, Wei-Chou Chang, Wu-Chien Chien, Ping-Ying Chang

**Affiliations:** 1 Department of Internal Medicine, Tri-Service General Hospital, National Defense Medical Center, Taipei City 11490, Taiwan; chenpohuang@hotmail.com; 2 Department of Neurology, Changhua Christian Hospital, Changhua City 500, Taiwan; xsai4295@gmail.com; 3 School of Public Health, National Defense Medical Center, Taipei City 11490, Taiwan; g694810042@gmail.com; 4 Department of Medical Research, Tri-Service General Hospital, National Defense Medical Center, Taipei City 11490, Taiwan; 5 Taiwanese Injury Prevention and Safety Promotion Association, Taipei City 11490, Taiwan; 6 Division of Hematology and Oncology Medicine, Department of Internal Medicine, Tri-Service General Hospital, National Defense Medical Center, Taipei City 11490, Taiwan; drleechohao@gmail.com (C.-H.L.); doc10456@mail.ndmctsgh.edu.tw (Y.-Y.W.); 7 Department of Radiology, Tri-Service General Hospital, National Defense Medical Center, Taipei City 11490, Taiwan; weichou.chang@gmail.com; 8 Graduate Institute of Life Sciences, National Defense Medical Center, Taipei City 11490, Taiwan

**Keywords:** statin, cancer risk, chronic kidney disease, dialysis

## Abstract

**Simple Summary:**

The lifetime risk of several cancers is elevated in patients receiving dialysis following kidney failure compared with the general population. Using a large dataset available in Taiwan, we conducted a nationwide population-based cohort study to delineate the relationship between statin use and cancer risk in patients on dialysis. Our study provides an association that statins reduce the risk of malignancy in patients on dialysis, especially with a longer treatment duration, and irrespective of the type of statin prescription. The use of statins in patients on dialysis was associated with significantly lower incidences in developing respiratory, soft tissue and connective tissue, breast, gynecological, prostate, central nervous system, and lymphatic and hematopoietic cancer.

**Abstract:**

Background: To realize whether statins reduce the risk of cancer in susceptible dialysis populations, this study analyzed the relationship between statin use and cancer risk in patients on dialysis. Methods: Patients having a history of chronic kidney disease with hemodialysis or peritoneal dialysis and receiving statin prescriptions or not were enrolled. The main outcome was cancer diagnosis. This study used univariate and multivariate Cox regression analyses. Results: In total, 4236 individuals in the statin group and 8472 individuals in the statin nonuser group were included in the study. Multivariate Cox regression analysis revealed that statin users are significantly less likely to develop cancer than statin nonusers (adjusted hazard ratio (HR) 0.81, 95% confidence interval (CI) 0.78–0.90). Subgroup analyses revealed that statin cumulative defined daily doses >365 were associated with a significantly decreased risk of cancer incidence (adjusted HR 0.59, 95% CI 0.45–0.87), and statin users have a reduced risk of respiratory, soft tissue and connective tissue, breast, gynecological, prostate, central nervous system, and lymphatic and hematopoietic cancer than nonusers. Conclusions: Our population-based cohort study provides an association that statins reduce the risk of malignancy in patients on dialysis, especially with a longer treatment duration, and certain types of cancer.

## 1. Introduction

End-stage renal disease (ESRD), which is the final permanent stage of chronic kidney disease, is a global health problem and life-threatening without proper renal replacement therapy (RRT) intervention. RRTs include kidney transplantation and dialysis, of which hemodialysis (HD) is the most common form of intervention in most countries. In 2015 alone, 83,808 patients in Taiwan received dialysis treatment [1]. However, patients on dialysis have an increased risk of malignancy [2]. A population-based cohort study conducted in northeastern Italy showed that the cumulative cancer risk of patients on dialysis was approximately 14% after 10 years of follow-up, corresponding to an overall 1.3-fold higher risk of de novo malignancies compared to the general population [3].

Statins decrease plasma lipid levels by inhibiting hydroxymethylglutaryl-CoA reductase (HMGCR). The addition of statin therapy also shows positive effects in the secondary prevention of ischemic stroke [4]. Statins are among drugs known to possess apoptosis-inducing effects and inhibit tumorigenesis; thus, statins exert a protective effect by reducing the risk of development of several types of cancer [5,6]. Observational studies have found that the use of statins before cancer diagnosis is associated with a lower risk of non-Hodgkin lymphoma, hepatocellular carcinoma, esophageal adenocarcinoma, and brain cancer [7,8,9,10]. Furthermore, a recent meta-analysis of 26 studies performed on more than 3 million participants and 170,000 patients with pancreatic cancer revealed a significant decrease in pancreatic cancer risk with statin use [11]. However, another meta-analysis of 14 other studies showed that statins had no effect on prostate cancer risk, whereas a different meta-analysis that included 32 studies involving 83,919 patients reported no significant association between statin use and breast cancer risk [12,13]. In summary, despite reports that statins reduce cancer risk, the results of various studies have been contradictory.

The lifetime risk of several cancers is elevated in patients receiving dialysis following kidney failure compared with the general population, highlighting the excess burden of cancer incidence in this vulnerable population [14]. Thus, there is an urgent need to identify specific medications associated with a lower risk of cancer incidence in patients on dialysis. However, whether statins reduce the risk of cancer in susceptible dialysis populations remains unknown. Using a large dataset available in Taiwan, we conducted a nationwide population-based cohort study to delineate the relationship between statin use and cancer risk in patients on dialysis.

## 2. Patients and Methods

### 2.1. Data Sources

A nationwide population-based study was conducted using data from 2000 to 2013 from the Longitudinal Health Insurance Database (LHID) of Taiwan. The LHID is a subset of the National Health Insurance Research Database (NHIRD) that is managed by the Taiwanese National Health Research Institutions. It enrolled one million randomly sampled beneficiaries from the NHIRD registry, which covers 99% of the Taiwanese population and provides medical services for 23 million residents. The LHID contains all information concerning sociodemographic information, medical visits, emergency care, hospitalization, surgical procedure, medication, as well as other medical services. Disease diagnoses were according to International Classification of Diseases, Ninth Revision, Clinical Modification (ICD-9-CM) codes. Previous studies have revealed that this database is suitable for use in pharmacoepidemiologic research. The Registry for Catastrophic Illness Patient Database (RCIPD) includes data from insured residents defined by the NHI program with severe diseases, such as malignancies and dialysis. This study was approved by the Institutional Review Board of the Tri-Service General Hospital, Taipei, Taiwan (TSGHIRB No. B-110-12). Because the data from NHI were deidentified, signed informed consent of included patients was waived.

### 2.2. Study Population and Definition of Statin Exposure

We extracted data from the LHID for patients who met the following criteria: (1) over 20 years of age with complete sex information, (2) a history of chronic kidney disease (ICD-9-CM 585) with hemodialysis (HD, ICD-9-CM Procedure 39.95) or peritoneal dialysis (PD, ICD-9-CM Procedure 54.98), and 3) disease diagnosis confirmed with RCIPD data from January 2000 through December 2013 longitudinally (Table S1). Patients with a diagnosis of malignancies (ICD9-CM Codes 140–209, Table S1) before tracing or tracing less than one year were excluded. The index date was defined as the date one year before the date of malignancy diagnosis. A group of control patients who were two-fold matched with the statin group was selected as controls, based on age (each 5-year span), sex, and index date year. The statin group was defined as patients who underwent statin therapy for at least 28 prescriptions of cumulative defined daily doses (cDDDs) of statin between cohort entry and index date before enrollment.

Complete information about all statin prescriptions was extracted from the registry for drug prescription database, which is also part of NHIRD. Data collected included the date of prescription, daily dose, and number of days supplied. For the statin treatment group, we calculated the total dosage prescribed during the follow-up period. We calculated cDDDs from prescription data recommended by the World Health Organization to measure the prescribed drug amount. DDD is the assumed average maintenance daily dose of a drug consumed for its main indication in adults [14]. To measure the dose–response relationship, patients were categorized into the following four groups according to the cDDD of statins used in the overall follow-up interval: less than 28 cDDD (nonuser), 28–90 cDDD, 91–365 cDDD, and greater than 365 cDDD.

### 2.3. Comorbidities

The following baseline comorbidities were identified: hypertension (ICD-9-CM Codes 401–405), diabetes mellitus (ICD-9-CM Code 250), chronic obstructive pulmonary disease (ICD-9-CM Codes 490–496), coronary artery disease (ICD-9-CM Codes 410–414), cerebrovascular accident (ICD-9-CM Codes 430–438), and liver cirrhosis (ICD-9-CM Codes 571.2, 571.5, 571.6, 572.2–572.4, 572.8, and 573.0). The overall Charlson Comorbidity Index Removed cancer (CCI_R) represented other less common comorbidities (Table S1) [15]. Medications reported having an impact on cancer development were enrolled, including thiazolidinedione (pioglitazone and rosiglitazone), and nonselective beta-blockers (propranolol and carvedilol) [16,17]. Other lipid-lowering drugs were also listed as confounding medications, including clofibrate, bezafibrate, gemfibrozil, fenofibrate, nicotinic acid, and acipimox (Table S1).

### 2.4. Study Outcome

The main outcome was cancer diagnosis. The confirmation of cancer (ICD-9-CM Codes 140–209) events was based on RCIPD data from the NHIRD. Histological and pathological confirmation of cancer was required for each patient. All subjects were followed from the index date until cancer occurrence, the date of withdrawal from the insurance system, or the end of 2013.

Three additional analyses were conducted to ascertain the association between statin and cancer incidence. We determined whether there was an association between statin dose effect analysis or different kinds of statins and cancer incidence, as well as whether statins were associated with the incidence of different types of cancer.

### 2.5. Statistical Analysis

Categorical variables were expressed using numbers (percentages) and continuous variables as mean ± standard deviation (SD). The Chi-square test was used for categorical variables, whereas Student’s t-test was used to compare the mean difference for continuous variables between statin users and the nonstatin group. Two-fold case-control matching was conducted using the variables of age, sex, and index date. The McNemar’s test was performed for categorical variables and the paired t-test or Wilcoxon signed-rank test was performed for continuous variables. Univariate and multivariate Cox regression analyses were employed to evaluate the adjusted hazard ratios (HRs) for the influence (odds) of the analyzed variables on developing cancer. Two-sided *p*-values < 0.05 were considered statistically significant. Kaplan–Meier analysis was performed to estimate the development of cancer in these two cohorts. All statistical analyses were performed using IBM SPSS Statistics for Windows version 22.0 (IBM Corp., Armonk, NY, USA).

## 3. Results

**Figure 1:**
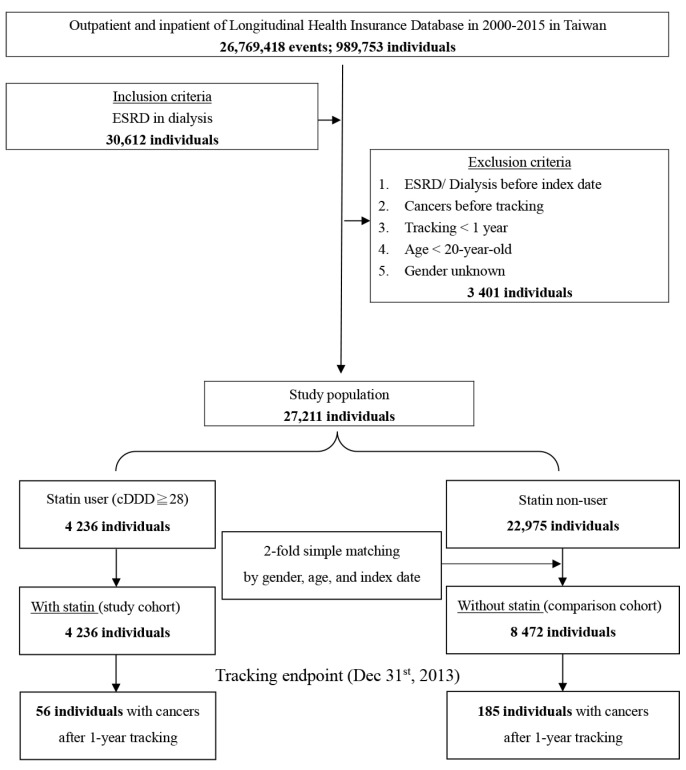
Flowchart of the study sample selection from the NHIRD in Taiwan cDDD = cumulative defined daily doses.

Among the 27,211 patients observed in this study, 4236 individuals were enrolled in the statin group and 22,975 individuals were controls in the statin nonuser group. After two-fold matching, there were 4236 individuals in the study cohort (statin group) and 8472 individuals in the comparison cohort (statin nonuser group). Figure 1 shows the overall workflow indicating how cases and controls were drawn from population databases, including the exclusion criteria used for both groups.

### 3.1. Patient Characteristics

Table 1 shows the demographics of our study population. After matching, the statin group had more women than men and the mean age ± SD of statin users and nonusers was 67.58 ± 16.76 and 67.67 ± 17.12 years, respectively, and the corresponding mean follow-up periods were 8.25 ± 9.45 and 8.31 ± 10.25 years. Table 1 also shows similar distributions due to well-balanced patient characteristics regarding the comorbidity burden and medication use between the two groups, except hypertension and CCI_R score.

### 3.2. Primary Outcomes

Among statin users and nonusers of patients on dialysis, after adjusting for age, gender, comorbidity (diabetes mellitus, hypertension, coronary artery disease, cerebrovascular accident, chronic obstruction pulmonary disease, and liver cirrhosis), CCI_R score, and medication (thiazolidinedione, nonselective beta-blocker, and other lipid-lowering drugs), multivariate analysis using Cox regression revealed that statin users were significantly less likely to develop cancer than statin nonusers (adjusted HR 0.81, 95% confidence interval (CI) 0.78–0.90, Table 2). We also determined that male (adjusted HR 1.24, 95% CI 1.03–1.60), middle age (45–64 years) (adjusted HR 2.79, 95% CI 1.68–4.65), elderly (>65 years) (adjusted HR 3.00, 95% CI 1.83–4.92), diabetes mellitus (adjusted HR 1.71, 95% CI 1.05–2.16), hypertension (adjusted HR 1.64, 95% CI 1.52–1.93), liver cirrhosis (adjusted HR 4.25, 95% CI 2.08–9.09), higher CCI_R score (adjusted HR 1.54, 95% CI 1.15–1.92), and the use of thiazolidinedione (adjusted HR 1.43, 95% CI 1.01–1.78) were independent risk factors regarding cancer incidence in patients on dialysis (Table 2).

### 3.3. Subgroup Analysis

To determine whether there was an association between dose effect analysis of statin on the risk of cancer incidence, we evaluated the effects of accumulated doses on cancer risk using cDDD. We found that a statin cDDD > 365 was associated with a significantly decreased risk of cancer incidence (adjusted HR 0.59, 95% CI 0.45–0.87, Table 3) compared with statin nonusers of patients on dialysis.

Next, we performed subgroup analysis to investigate different subtypes of statin with cancer risk, and found that all statin subtypes consistently revealed a decreased risk for cancer incidence compared with statin nonusers (Table 3).

In addition, we analyzed the association between statin prescription and different types of cancer. Our results revealed that the use of statins in patients on dialysis was associated with a significantly lower incidence in developing respiratory (adjusted HR 0.53, 95% CI 0.50–0.60), soft tissue and connective tissue (adjusted HR 0.45, 95% CI 0.42–0.50), breast (adjusted HR 0.47, 95% CI 0.44–0.53), gynecological (adjusted HR 0.78, 95% CI 0.42–0.95), prostate (adjusted HR 0.53, 95% CI 0.51–0.59), central nervous system (adjusted HR 0.89, 95% CI 0.72–0.998), as well as lymphatic and hematopoietic (adjusted HR 0.67, 95% CI 0.64–0.75) cancer compared with statin nonusers (Table 4).

### 3.4. Kaplan–Meier Plot for Cumulative Cancer Incidence

**Figure 2:**
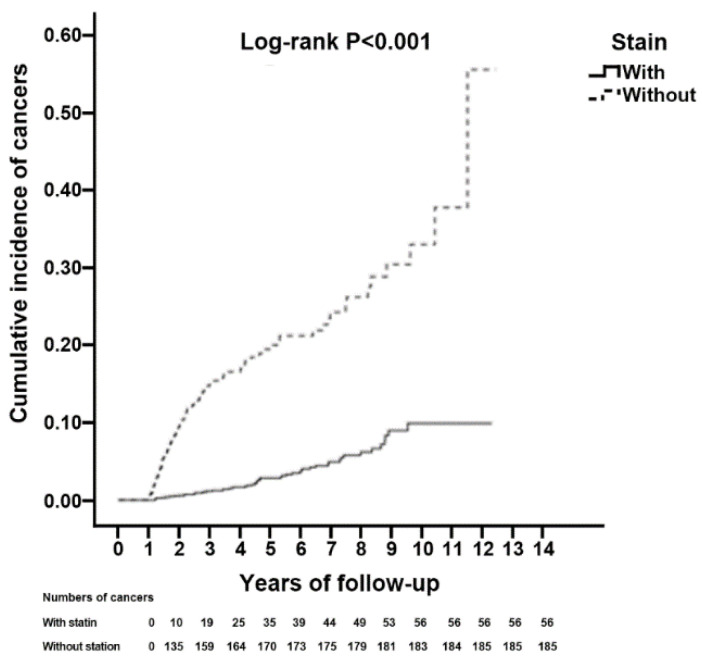
Kaplan–Meier for cumulative incidence of cancers after 1-year tracking among ESRD in dialysis patients aged 20 and over stratified by Statin with the log-rank test.

The incidence of cancer during the follow-up period was illustrated through a Kaplan–Meier plot. The results showed a lower cumulative incidence rate of cancers in those prescribed statins compared with patients not prescribed statins (Log-rank test, *p* value < 0.001, Figure 2) and the corresponding periods until the first diagnosis of cancers were 4.62 ± 2.47 and 2.20 ± 1.90 years.

## 4. Discussion

### 4.1. Major Findings

Our study is the first large-scale real-world study exploring the association between the use of statins and risk of cancer incidence in patients on dialysis. Our results revealed a lower risk of cancer incidence in patients on dialysis using statins compared to those not using statins, irrespective of the type of statin prescription. Furthermore, we identified a dose–response effect of statin use on cancer risk. The use of statins in patients on dialysis was associated with significantly lower incidences in developing respiratory, soft tissue and connective tissue, breast, gynecological, prostate, central nervous system, and lymphatic and hematopoietic cancer.

### 4.2. Biological Plausibility

There are several convincing biological explanations of how statin use reduces cancer risk. Indeed, statins exert several pleiotropic effects to reduce the risk of cancer, including improving endothelial function, decreasing vascular inflammation, and inhibiting smooth-muscle proliferation, as well as antioxidative and anti-inflammatory effects [18]. Statins are widely prescribed inhibitors of the mevalonate pathway, acting to lower systemic cholesterol levels. Recently, there have been many reports linking upregulated activity of mevalonate and downstream metabolic pathways to cancer development and progression [19]. For example, in breast cancer, it was found that high levels of HMGCR and other mevalonate pathway gene transcripts correlated with poor prognosis. HMGCR is targeted by statins in breast cancer cells in vivo, and statins have an antiproliferative effect in HMGCR-positive tumors [20]. Further, statins activate the expression of the onco-suppressor miRNA-145, which controls tumor cell migration and invasion eventually [21].

### 4.3. The Effect of Statins in Patients on Dialysis

Previous studies have discussed the impact of statins in patients on dialysis mainly focused on cardiovascular events and overall mortality. The AURORA trial, which was a randomized placebo-controlled trial, failed to reveal any beneficial effect of statins on cardiovascular outcomes in patients receiving dialysis and also reported that all-cause mortality was unaffected by statin treatment [22]. However, the Study of Heart and Renal Protection showed that moderate-intensity statin therapy in combination with ezetimibe reduced the risk of major atherosclerotic events in patients on dialysis [23]. Another large multicenter randomized controlled trial, the Der Deutsche Diabetes Dialyse (4D) study, revealed that atorvastatin treatment in patients with type 2 diabetes on maintenance dialysis treatment does not improve cardiovascular outcomes. However, the latest large retrospective cohort study by Jung et al. revealed that statin therapy in patients on maintenance dialysis was associated with a lower risk of all-cause mortality [24]. To date, the findings of studies examining the cardiovascular effects of statin use in patients on dialysis have been inconsistent.

However, a Japanese study investigated the effects of statins in Asians undergoing maintenance hemodialysis, and suggested statins reduce mortality due to cardiovascular events, infections, and cancer [25]. Moreover, they proposed that statins may influence cancer risk reduction, although their results came from a small number of participants. Our data from a larger population and more statistically robust results showed a consistent consequence. Additionally, although Kidney Disease Outcomes Quality Initiative guidelines recommend that statin therapy should not be initiated in patients with type 2 diabetes on maintenance dialysis without specific cardiovascular indications for treatment, other important roles of statins in patients on dialysis emerged in our study, namely, that statins reduce cancer incidence in dialysis settings [26].

### 4.4. Effect of Different Statins

Although all statins inhibit HMGCR activity in extrahepatic tumor tissues, differences exist between different types of statins. It was hypothesized that lipophilic statin drugs are more likely to reach and readily enter extrahepatic cells, whereas hydrophilic statins are more hepatoselective in the liver [27]. A previous epidemiological study reported that use of lipophilic statins (atorvastatin, simvastatin, lovastatin, fluvastatin, and pitavastatin), but not hydrophilic statins (rosuvastatin and pravastatin), is associated with reduced cancer incidence [28]. However, a network meta-analysis of seven studies focusing on statin use and cancer incidence in patients with type 2 diabetes mellitus concluded that the hydrophilic statin rosuvastatin was most effective, followed by fluvastatin and atorvastatin [29]. In our study, we found that all types of statins (lipophilic and hydrophilic) reduced the risk of cancer among patients on dialysis.

### 4.5. Statins and Risk of Cancer Types

In the general population, an umbrella systematic review and meta-analysis conducted by Jeong et al. analyzed the data of 43 meta-analyses of randomized controlled trials and observational studies on associations between statin use and cancer incidence, and found that statins have a statistically significant effect on reducing cancer incidence in 10 of 18 types of cancer [30]. However, after grading the level of evidence, the umbrella meta-analysis claimed that only four cancers (esophageal cancer, hematological cancer, leukemia, and liver cancer) had suggestive evidence of a preventive effect, and there was only weak evidence for the remaining six cancer types. As findings regarding the effect of statins in reducing the risk of cancer are conflicting, evidence in more specific patient characteristics and risk of incidence in different cancer types is warranted. Among the four cancers (esophageal cancer, hematological cancer, leukemia, and liver cancer) with suggestive evidence in the umbrella meta-analysis, we report similar results that statins reduce the incidence of hematopoietic cancers [30].

Another umbrella review of meta-analyses, published earlier, revealed that statins showed health benefits in reduced risk of hematologic, liver, gastric, colorectal, esophageal, and prostate cancer in the overall population based on combined meta-analyses of observational studies; however, only weak evidence was assessed [31]. A different meta-analysis of observational studies of 14 studies revealed that statin use, compared to the nonuse of statins, was negatively associated with all hematological malignancies, taken together (relative risk 0.86, 95% CI 0.77–0.96) [32]. In particular, long-term statin users have a statistically significant reduction in the risk of all hematological malignancies (relative risk 0.78, 95% CI 0.71–0.87). In our population-level cohort study, we investigated statin use and cancer risk in patients receiving dialysis but also performed subgroup analyses to evaluate the occurrence of different types of cancer. Based on our results, statins have credible effects on reducing the development of hematopoietic cancers regardless of the dialysis population.

### 4.6. Other Lipid-Lowering Drugs and Cancer Prevention

Basic studies have shown that high cholesterol might lead to cancer development [33], and continuous cholesterol supply was necessary for highly proliferating cancer cells in membrane biogenesis [34]. Fibrates are peroxisome proliferator-activated receptor (PPAR) agonists frequently prescribed to treat hypertriglyceridemia that may also affect carcinogenesis [35]. A previous meta-analysis showed that fibrates had a neutral effect on cancer incidence [36]. However, the latest study also revealed that fibrates treatment might be associated with reduced long-term cancer incidence among patients with coronary artery disease. Emerging studies have discussed the role of available lipid-lowering drugs in cancer incidence and treatment [37]. Our data showed an insight that patient with lipid-lowering drugs, especially fibrates, might have a lower risk in cancer development, compared to those without. However, further large-scale prospective studies are warranted.

### 4.7. Strengths and Limitations

The strengths of our study include the use of a large study population on a nationwide scale with a continuously updated high-quality claim database, consisting of a large number of adults with dialysis-dependent chronic kidney disease, and the use of records from the NHIRD that covered all patients with ESRD and whose diagnosis was confirmed by RCIPD in Taiwan during the study period. Furthermore, we required a minimum of 28 prescriptions to be defined as a “statin user”, thereby minimizing the possibility that patients filled a prescription without taking the drug, and we specifically adjusted for concomitant drug use reported to impact cancer development.

This study has a few limitations. First, we endeavored to adjust the most common risk factors for cancer development, although not all variables associated with cancer development were available in our study, such as smoking history, some types of viral infections, specific chemicals, or radiation exposure. The NHIRD data do not provide information pertaining to laboratory data, lifestyle, or family history that may be related to cancer incidence. Second, the severity or stage of comorbidity was not adequately captured, although we used CCI_R for adjustments. Third, although cancer development should be diagnosed by pathology reports, we were unable to obtain the results of pathology reports from the database. As we could not retrieve the exact time point of cancer onset, we included only patients who were definitively diagnosed with cancer using the registry of catastrophic illness (RCIPD) to ensure the accuracy of cancer diagnosis. Fourth, most of our study population was Asian, and thus, additional studies in a more ethnically diverse cohort are warranted.

## 5. Conclusions

Our population-based cohort study provides an association that statins reduce the risk of malignancy in patients on dialysis, especially with a longer treatment duration, and irrespective of the type of statin prescription. We also found that statin users have a statistically significantly reduced risk of respiratory, soft tissue and connective tissue, breast, gynecological, prostate, central nervous system, and lymphatic and hematopoietic cancer than nonusers.

## Figures and Tables

**Table 1:** Characteristics of the study at baseline.

Statin	Total		With		Without		
Variables	*n*	%	*n*	%	*n*	%	*p*
**Total**	12,708		4236	33.33	8472	66.67	
**Year of follow-up**	8.29 ± 9.83		8.25 ± 9.45		8.31 ± 10.25		
**Gender**							0.999
Male	6285	49.46	2095	49.46	4190	49.46	
Female	6423	50.54	2141	50.54	4282	50.54	
**Age (years)**	67.64 ± 17.00		67.58 ± 16.76		67.67 ± 17.12		0.779
**Age groups (years)**							0.999
20–44	1017	8.00	339	8.00	678	8.00	
45–64	4395	34.58	1465	34.58	2930	34.58	
≧65	7296	57.41	2432	57.41	4864	57.41	
**Diabetes Mellitus**							0.111
Without	8704	68.49	2862	67.56	5842	68.96	
With	4004	31.51	1374	32.44	2630	31.04	
**Hypertension**							<0.001
Without	9457	74.42	3040	71.77	6417	75.74	
With	3251	25.58	1196	28.23	2055	24.26	
**Coronary artery disease**							0.578
Without	12,333	97.05	4106	96.93	8227	97.11	
With	375	2.95	130	3.07	245	2.89	
**Cerebrovascular accident**							0.638
Without	12,250	96.40	4088	96.51	8162	96.34	
With	458	3.60	148	3.49	310	3.66	
**Chronic obstructive pulmonary disease**							0.112
Without	11,921	93.81	3994	94.29	7927	93.57	
With	787	6.19	242	5.71	545	6.43	
**Liver cirrhosis**							0.739
Without	12,024	94.62	4004	94.52	8020	94.66	
With	684	5.38	232	5.48	452	5.34	
**Charlson Comorbidity Index Removed cancer**	0.36 ± 0.63		0.38 ± 0.65		0.35 ± 0.62		0.011
**Thiazolidinedione**							0.230
Without	9003	70.85	2972	70.16	6031	71.19	
With	3705	29.15	1264	29.84	2441	28.81	
**Non-selective beta-blocker**							0.254
Without	9948	78.28	3291	77.69	6657	78.58	
With	2760	21.72	945	22.31	1815	21.42	
**Other lipid-lowering drugs**							0.302
Without	12,277	96.61	4102	96.84	8175	96.49	
With	431	3.39	134	3.16	297	3.51	

*p*: Chi-square/Fisher exact test on category variables and *t*-test on continous variables.

**Table 2:** Factors of cancers using Cox regression.

Variables	Crude HR	95% LCI	95% UCI	*p*	Adjusted HR	95% LCI	95% UCI	*p*
**Statin**								
Without	Reference				Reference			
With	0.765	0.612	0.892	<0.001	0.807	0.779	0.904	<0.001
**Gender**								
Male	1.684	1.145	2.564	0.003	1.235	1.030	1.599	0.019
Female	Reference				Reference			
**Age groups (yrs)**								
20–44	Reference				Reference			
45–64	3.121	1.897	5.124	<0.001	2.793	1.678	4.649	<0.001
≥65	3.675	2.044	5.682	<0.001	2.999	1.830	4.917	<0.001
**Diabetes Mellitus**								
Without	Reference				Reference			
With	1.985	1.124	3.826	<0.001	1.711	1.049	2.162	0.001
**Hypertension**								
Without	Reference				Reference			
With	2.684	1.501	4.565	<0.001	1.694	1.520	1.928	<0.001
**Coronary artery disease**								
Without	Reference				Reference			
With	1.422	0.562	3.267	0.678	1.395	0.502	3.221	0.577
**Cerebrovascular accident**								
Without	Reference				Reference			
With	1.201	0.331	3.186	0.594	1.143	0.303	3.106	0.681
**Chronic obstructive pulmonary disease**								
Without	Reference				Reference			
With	0.862	0.435	1.297	0.297	1.035	0.580	2.130	0.471
**Liver cirrhosis**								
Without	Reference				Reference			
With	5.234	2.672	19.762	<0.001	4.245	2.077	9.085	<0.001
**Charlson Comorbidity Index Removed cancer**	1.584	1.264	1.972	<0.001	1.543	1.152	1.925	<0.001
**Thiazolidinedione**								
Without	Reference				Reference			
With	1.672	1.184	2.044	0.007	1.428	1.010	1.781	0.038
**Non-selective beta-blocker**								
Without	Reference				Reference			
With	0.754	0.453	1.249	0.551	1.011	0.680	2.128	0.719
**Other lipid-lowering drugs**								
Without	Reference				Reference			
With	0.511	0.349	0.896	<0.001	0.772	0.485	0.994	0.045

HR = hazard ratio, LCI = confidence interval, UCI = upper confidence interval, Adjusted HR: Adjusted variables listed in the table.

**Table 3:** Factors of cancers stratified by Statin cDDD/subtype using Cox regression.

Model	Statin	Population	Events	PYs	Rate (Per 10^5^ PYs)	Adjusted HR	95% LCI	95% UCI	*p*
**Model 1**	Without	8472	185	79,575.9	232.48	Reference			
With/Without	With	4236	56	39,168.6	142.97	0.807	0.779	0.904	<0.001
**Model 2**	Without	8472	185	79,575.9	232.48	Reference			
cDDD	28–90 cDDD	2623	29	18,456.1	157.13	0.963	0.793	1.278	0.231
	91–365 cDDD	884	16	10,945.5	146.18	0.813	0.784	1.010	0.061
	>365 cDDD	729	11	9767.02	112.62	0.594	0.446	0.869	<0.001
**Model 3**	Without	8472	185	79,575.9	232.48	Reference			
Subtype	Simvastatin	612	8	5657.9	141.39	0.798	0.769	0.894	<0.001
	Fluvastatin	604	7	5530.0	126.58	0.715	0.690	0.801	<0.001
	Lovastatin	622	9	5776.4	155.81	0.879	0.843	0.985	0.036
	Atorvastatin	598	8	5531.5	144.63	0.815	0.780	0.913	<0.001
	Pravastatin	583	9	5926.6	151.85	0.854	0.821	0.961	0.010
	Rosuvastatin	617	8	5698.2	140.40	0.793	0.758	0.891	<0.001
	Pitavastatin	600	7	5048.0	138.67	0.782	0.745	0.877	<0.001

PYs = Person-years; Adjusted HR = Adjusted Hazard ratio: Adjusted for the variables listed in Table 2; LCI = confidence interval, UCI = upper confidence interval; cDDD = cumulative defined daily doses.

**Table 4:** Factors of cancers subgroups using Cox regression.

Statin	With			Without (Reference)				With vs. Without (Reference)			
Cancer Subgroup	Ets	PYs	Rate (per 10^5^ PYs)	Ets	PYs	Rate (per 10^5^ PYs)	Ratio	Adjusted HR	95%LCI	95%UCI	*p*
Total	56	39,168.6	142.97	185	79,575.9	232.48	0.615	0.807	0.779	0.904	<0.001
Oral cavity and pharynx	5	39,168.6	12.77	15	79,575.9	18.85	0.677	0.888	0.840	1.013	0.071
Digestive	18	39,168.6	45.96	48	79,575.9	60.32	0.762	1.000	0.928	1.122	0.157
Respiratory	4	39,168.6	10.21	20	79,575.9	25.13	0.406	0.533	0.501	0.598	<0.001
Soft tissue/connective tissue	1	39,168.6	2.55	6	79,575.9	7.54	0.339	0.445	0.423	0.502	<0.001
Breast	3	39,168.6	7.66	17	79,575.9	21.36	0.359	0.471	0.437	0.531	<0.001
Gynecological	2	39,168.6	5.11	11	79,575.9	13.82	0.369	0.783	0.423	0.946	<0.001
Prostate	1	39,168.6	2.55	5	79,575.9	6.28	0.406	0.532	0.508	0.590	<0.001
Urinary tract	18	39,168.6	45.96	38	79,575.9	47.75	0.962	1.261	0.959	1.444	0.288
Central nervous system	1	39,168.6	2.55	3	79,575.9	3.77	0.677	0.892	0.721	0.998	0.049
Lymphatic and hematopoietic	3	39,168.6	7.66	12	79,575.9	15.08	0.508	0.666	0.641	0.748	<0.001

Ets = events; PYs = Person-years; Adjusted HR = Adjusted Hazard ratio: Adjusted for the variables listed in Table 2; LCI = confidence interval, UCI = upper confidence interval.

## Data Availability

Data is contained within the article and Supplementary Material.

## Supplementary Materials

The following are available online at https://www.mdpi.com/article/10.3390/jcm10235602/s1, Table S1: Abbreviation, ICD-9-CM and definition.
