# Supplementary material for: The Effect of Statins in Cancer Risk Reduction in Patients on Dialysis: A Population-Based Case-Control Study
Source: J Clin Med. 2021 Nov 28;10(23):5602. doi: 10.3390/jcm10235602 (PMC8658442; doi:10.3390/jcm10235602)
Supplement: Supplementary file 1 [file jcm-10-05602-s001.zip › jcm-1489690-supplementary.pdf]

Supplementary Information

The Effect of Statins in Cancer Risk Reduction in Patients on Dialysis: A Population-based Case-Control Study

Po-Huang Chen[1], Hong-Jie Jhou[2#], Chi-Hsiang Chung[3,4,5], Cho-Hao Lee[6], Yi-Ying Wu[6], Wei-Chou Chang[7], Wu-Chien Chien[3,4,8*], Ping-Ying Chang[6*]

Contents

Supplementary Information 1. Abbreviation, ICD-9-CM and definition

Information 1 Abbreviation, ICD-9-CM and definition

Table S1. Abbreviation, ICD-9-CM and definition.

	Abbreviation	ICD-9-CM / Definition
**Study population:**		≧ 3 outpatients in 6 months or inpatient
End-stage renal disease	ESRD	585, with catastrophic illness card
Dialysis		Any of the following
Hemodialysis	HD	OP39.95
Peritoneal hemodialysis	PD	OP54.98
**Events: Cancer**		140-209, with catastrophic illness card
Oral cavity and pharynx		140-149
Digestive		150-159
Respiratory		160-165
Soft tissue / connective tissue		170-171, 173
Breast		174-175
Gynecological		179-184
Prostate		185
Urinary tract		188-189
Central nervous system		191-192
Lymphatic and hematopoietic		200-209
**Intervention: Statin**		Simvastatin, Fluvastatin, Lovastatin, Atorvastatin, Pravastatin, Rosuvastatin, and Pitavastatin
**Comorbidities**		≧ 2 outpatients or inpatient within 6 months before and after the index date
Diabetes mellitus	DM	250
Hypertension	HTN	401-405
Coronary artery disease	CAD	410-414
Cerebrovascular accident	CVA	430-438
Chronic obstructive pulmonary disease	COPD	490-496
Liver cirrhosis		571.2, 571.5-571.6, 572.2-572.4, 572.8, 573.0
**Charlson comorbidity index revised**	CCI_R	CCI removed CKD, cancers, DM, HTN, CAD, CVA, COPD, and liver disease
